# Temporal Variation of SARS-CoV-2 Levels in Wastewater from a Meat Processing Plant

**DOI:** 10.3390/microorganisms11010174

**Published:** 2023-01-10

**Authors:** Meiyi Zhang, Maria D. King

**Affiliations:** Aerosol Technology Laboratory, Biological & Agricultural Engineering Department, Texas A&M University, College Station, TX 77843, USA

**Keywords:** wastewater-based surveillance, SARS-CoV-2, RT-qPCR, meat processing plants

## Abstract

Wastewater-based surveillance (WBS) on SARS-CoV-2 has been proved to be an effective approach to estimate the prevalence of COVID-19 in communities and cities. However, its application was overlooked at smaller scale, such as a single facility. Meat processing plants are hotspots for COVID-19 outbreaks due to their unique environment that are favorable for the survival and persistence of SARS-CoV-2. This is the first known WBS study in meat processing plants. The goal was to understand the temporal variation of the SARS-CoV-2 levels in wastewater from a meat processing plant in Canada during a three-month campaign and to find any correlation with clinically confirmed cases in the surrounding city area. Higher SARS-CoV-2 concentrations and detection frequencies were observed in the solid fraction compared to the liquid fraction of the wastewater. The viruses can be preserved in the solid fraction of wastewater for up to 12 days. The wastewater virus level did not correlate to the city-wide COVID-19 cases due to the unmatching scales. WBS on SARS-CoV-2 in meat processing plants can be useful for identifying COVID-19 outbreaks in the facility and serve as an effective alternative when resources for routine individual testing are not available.

## 1. Introduction

Wastewater-based epidemiology (WBE) has been extensively studied since the COVID-19 pandemic and has proved to be a critical tool for COVID-19 monitoring, especially useful for regions where public health resources are not readily available. One of the most important advantages of WBE in studying the prevalence of pathogen, including SARS-CoV-2, among the population is that the sources of the viruses in the wastewater contain both symptomatic and asymptomatic individuals, which makes it a promising tool to capture the asymptomatic patients who otherwise do not participate in clinical testing. Most WBS studies for COVID-19 focused on analyzing and monitoring wastewater samples taken from wastewater treatment plants (WWTPs) or hospitals [1,2,3,4]. Several studies showed a correlation between SARS-CoV-2 levels in wastewater and positive COVID-19 cases reported from clinic testing or public records, suggesting that wastewater-based monitoring can provide information for large-scale dynamic of the pandemic [5,6,7,8,9]. Although it is important to investigate the COVID-19 prevalence and dynamic in communities and cities scales, with the negative economic impact of the COVID-19 around the world, the interests of business owners lay in the presence of the viruses and infected individuals in the local facility, which is crucial for updating the control procedures in a timely manner to respond to the decreasing or rising risks and ensuring the safety of the workers during the business recovery stage [10,11,12].

Meat processing plants are critical infrastructure that have been identified as one of the hotspots for COVID-19 outbreaks [13,14,15,16,17]. A group of researchers conducted a retrospective investigation on a plant with 111 confirmed asymptomatic cases with an attack rate of 38% during a five-week period after the first case and showed that the area with the highest infection rate, the boning hall, had poor ventilation and a favorable environment for viral aerosol transmission [13]. Fabreau et al. [14] used meat processing plants as a model to study the COVID-19 risk among migrant populations and concluded that meat processing plants are at high risks for COVID-19 transmission due to the noisy and confined workspaces where workers are often physically demanded and require shouting for communication, thus increasing the number of aerosolized droplets and pathogen transmission risk. Mallet et al. [15] also identified the deboning and cutting area of a French meat processing plant as highly vulnerable to COVID-19 outbreaks and revealed an increased risk of infection for foreign-born workers who were more likely to share accommodation and carpooling. Other studies performed in meat processing plants found a correlation between infection risk and indoor climate conditions, indoor ventilation, outdoor air flow rate, high density of workers, and prolonged contact in the facility [16,17,18].

This is the first known study to show the dynamic changes in virus concentrations in meat processing plants, which often contain a unique environment—low temperature, high RH, high fat and protein content in the air—that was shown by a previous study of the authors to favor the attachment and preservation of SARS-CoV-2 viruses [19]. A three-month campaign was launched in early 2022 to monitor the wastewater samples collected from a meat processing plant in Canada by detecting and quantifying the SARS-CoV-2 viruses using RT-qPCR to understand the temporal variation of the virus levels over the project period and to find any correlation between the virus levels in wastewater and clinically confirmed cases in the surrounding city area.

## 2. Materials and Methods

### 2.1. Wastewater Sampling and Processing

Wastewater was collected on site by meat processing plant personnel from the tank (plant waste) and pit (human waste) from 11 January to 3 April 2022. Wastewater samples were collected in sterile 50 mL centrifuge tubes or clear plastic bottles, and stored in a freezer before shipping to the laboratory for analysis. Upon delivery, samples were stored at 4 °C overnight to allow sedimentation of the solid particles. Each sample was separated into liquid and sludge fractions and vortexed until mixed thoroughly. The clear supernatant from each fraction was diluted 10 times in Phosphate Saline Buffer (PBS) to reduce the impact of any inhibitors that might be present in the wastewater samples.

### 2.2. RT-qPCR Analysis

RT-qPCR was performed directly to quantify the SARS-CoV-2 in the wastewater samples with the premixed primer and probes set 2019-nCoV RUO Kit (Integrated DNA Technologies, Inc., Coralville, IA, USA). A combination of the N1 and N2 primers and probes set was chosen because a study showed that this duplex qPCR assay increased the sensitivity of SARS-CoV-2 detection in wastewater [20]. The sequences of the primers and probes were found in the study of Qiu et al. [21]. Each reaction contains the wastewater sample as the template, N1 and N2 primers/probes, and the PowerSYBR Green PCR Master Mix (Applied Biosystems, Warrington, UK). The amplification process was performed with the AB StepOne RT-PCR System (Applied Biosystems, Foster City, CA, USA). Samples were considered positive if amplification was achieved within a cycle threshold of less than 40 cycles (Ct < 40) for at least two out of the triplicates. Dilutions of the 2019nCoV_N Positive Control (Integrated DNA Technologies, Inc., Coralville, IA, USA) were prepared in triplicates to generate a standard curve that was used to calculate the gene copy number (GCN) of SARS-CoV-2.

### 2.3. COVID-19 Cases Information from the Surrounding City

A daily increase of clinically diagnosed COVID-19 cases from Alberta was obtained from the public database of the Government of Alberta (Government of Alberta, 2022). The information about the confirmed cases during the sample collection period from the zone where the meat processing plant of interest belonged was extracted using RStudio 2021.09.2+382.

### 2.4. Statistical Analysis

The average and standard deviation of SARS-CoV-2 concentrations were computed and plotted on graphs. To measure the linear correlation between the SARS-CoV-2 concentrations in wastewater and the new COVID-19 cases in adjacent city during the collection period, a Pearson’s correlation coefficient analysis was performed and *p*-value was calculated to show the statistical significance. A 5% level of significance was used to compare with the calculated *p*-value.

## 3. Results

This study initially included wastewater sampling from both a tank system (i.e., plant waste including the dirty water from hosing and washing the floor and workstations in the meat processing rooms) and a pit system (human waste or sewage). The study later focused only on the tank wastewater since all the pit wastewater failed to achieve detectable amplification (Ct > 40) and returned negative results. As a result, this paper only shows the results and analysis of the tank wastewater from the meat processing plant as further analysis on the pit wastewater would require substantial additional resources and time which are beyond the scope of this study.

**Figure 1:**
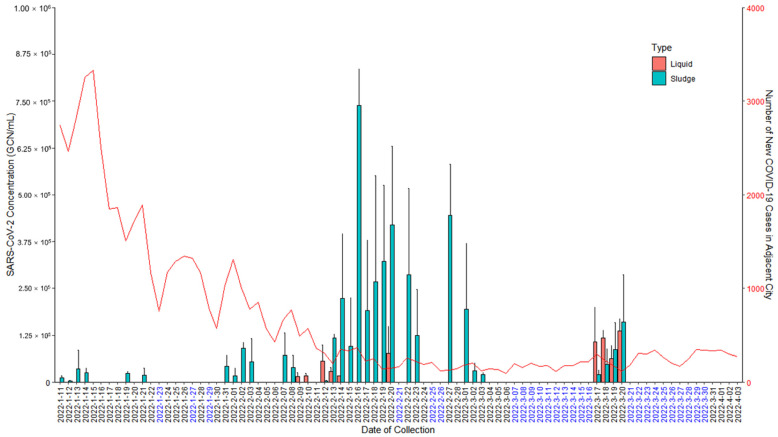
SARS-CoV-2 concentration in the liquid fraction (pink bar) and sludge fraction (green bar) of the tank wastewater from the meat processing plant from 11 January 2022 to 3 April 2022. The dates where wastewater was not collected are marked in blue. SARS-CoV-2 quantification results were obtained from qRT-PCR analysis with conversion from Ct values to GCN/mL based on the standard curve. The number of new clinically diagnosed COVID-19 cases on each date in the adjacent city area during the wastewater collection period is shown by the red curve.

Wastewater from the tank of the meat processing plant was sampled from a total of 57 days between 11 January 2022 to 3 April 2022. Each wastewater collection was separated into liquid and sludge fractions resulting in 57 liquid samples and 57 sludge samples, and analyzed separately for SARS-CoV-2 presence and quantification. Out of the 114 wastewater samples, 41 collections tested positive (35.96% positivity rate), including 10 liquid fractions (17.54% positivity rate from liquid samples) and 31 sludge fractions (54.39% positivity rate from sludge samples). The mean and median of SARS-CoV-2 Genome Copy Number (GCN) were 6.34 × 10^4^ and 5.99 × 10^4^ per mL liquid fraction of wastewater, and 1.76 × 10^5^ and 7.08 × 10^4^ per mL sludge fraction of wastewater, respectively. The wastewater sludge fractions had higher positivity rate and SARS-CoV-2 concentration compared to the wastewater liquid fractions. Figure 1 shows the SARS-CoV-2 quantification results from the liquid and sludge fractions of the tank wastewater from the meat processing plant from 11 January 2022 to 3 April 2022 where collections were conducted (date marked in black), as well as the number of new COVID-19 cases in the adjacent city on each date. The cluster of positive results from 13 February 2022 to 23 February 2022 also showed higher viral load and indicated that the virus might be preserved in the sludge for a prolonged time period of up to 12 days despites the wastewater treatment system installed.

Table 1 shows the detection of SARS-CoV-2 in the liquid fraction of the wastewater during the collection period. Out of the total of 83 days during the study campaign in 2022, positive wastewater was obtained from 10 days, negative wastewater was obtained from 47 days, and no information was provided due to missing collection from 26 days. Table 2 shows the SARS-CoV-2 detection in the sludge fraction of the wastewater during the same time period. Out of the 83 days from 11 January 2022 to 3 April 2022, the wastewater was tested positive for 31 days, negative for 26 days, and missing for 26 days. Information about the tank wastewater treatment system and schedule must be obtained in order to explain the virus detection and accumulation and evaluate the relationship between the positive wastewater and the treatment schedule of the current system. On every workday, ~750,000 L waste was generated and added in the tank, and ~600,000 L waste was treated, leaving a net increase of ~150,000L per workday. On Sunday, no waste was added, and after the treatment 30% of what was in the tank was left (300,000 L to 400,000 L) and carried over to the next Monday. The full capacity of ~4.5 million L was not expected to be reached in the tank, and there was no full turnover. Further details were restricted by the request of the meat processing plant.

**Figure 2:**
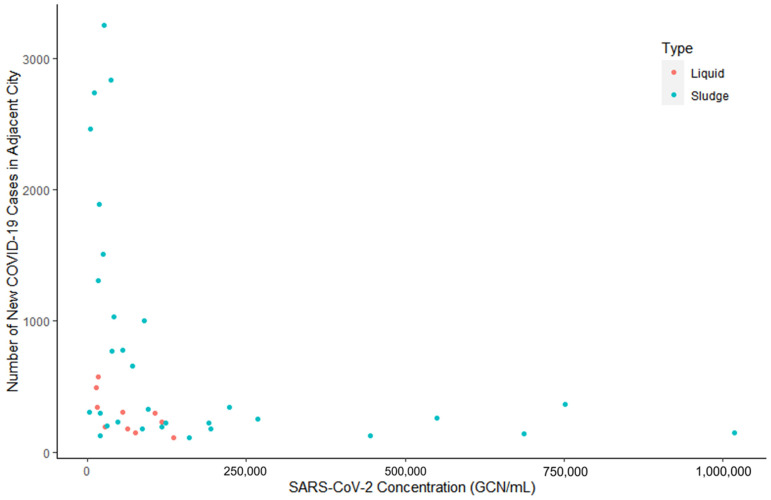
SARS-CoV-2 concentration (GCN/mL) from the positive wastewater in the liquid fraction (green) and sludge fraction (pink) and the number of new COVID-19 cases in adjacent city on the collection date.

Figure 2 shows the SARS-CoV-2 concentration (GCN/mL) from the positive wastewater in the liquid and sludge fraction and the number of new COVID-19 cases in adjacent city on the collection date. This distribution of data is right skewed as six positive wastewater samples with virus concentration greater than 250,000 GCN/mL were from dates where the number of new COVID-19 cases were low. These observations with high SARS-CoV-2 contents and low number of new COVID-19 cases were likely contributed by the continuous detection of positive wastewater sludge from mid-February. Overall, the correlation between SARS-CoV-2 contents and new COVID-19 cases was poor, if any. A Pearson’s correlation coefficient analysis was performed and showed that the correlation between SARS-CoV-2 concentrations in wastewater and the new COVID-19 cases in an adjacent city during the collection period failed to return a significant *p*-value (*p*-value = 0.07). The correlation is therefore not statistically significant at 5% level and there is no evidence that any correlation exists. This was expected because the wastewater was collected directly on site of the meat processing plant, and the publicly available COVID-19 information was extracted from the government website where the smallest scale of testing results was the city level. Although the workers at the meat processing plant live in the adjacent city or communities whose COVID-19 testing information was included in the database used in this study, the city population is much larger than the workers alone and may not closely reflect the situation in the meat processing plant.

## 4. Discussion

Wastewater-Based Surveillance (WBS) has been proved to be an effective approach that utilizes the level of SARS-CoV-2 in wastewater to estimate the prevalence and mitigation of COVID-19 in a large area, such as communities or cities. Acosta et al. [1] investigated the SARS-CoV-2 levels in hospital wastewater and found a correlation between the virus abundance and increasing hospitalizations. In a 3-month study of wastewater-based city zonation, the variation of SARS-CoV-2 RNA in the wastewater was shown to lead the change in the confirmed cases by 1-2 weeks, giving the public up to 2 weeks to prepare and manage for the pandemic situation in advance [22]. Pang et al. [9] showed that different correlations between the wastewater virus levels were observed depending on the sizes of communities, and concluded that the information obtained with WBS was unbiased compared to clinical testing which is highly dependent on the local policies. Another study in Brazil found a lead of 5 days in the detection of SARS-CoV-2 in wastewater compared to the reported positive cases [23]. Although the application of WBS has been evaluated in communities and cities where direct records of positive COVID-19 cases were available, the reliability of using COVID-19 public records, where the clinical testing results are obtained from community or city-scaled area, to estimate the prevalence of COVID-19 in a business such as a meat processing plant remains unclear. Nevertheless, such estimation is crucial for the safe reopening of business to restore the economy post-pandemic. Studies have showed that meat processing plants are under high risks of COVID-19 infections and higher probability of serious outbreaks and superspreading event [24,25,26]. This study aims to examine the application of WBS at a facility level by monitoring the SARS-CoV-2 levels in wastewater from a meat processing plant to understand the temporal variation and any correlation with the reported COVID-19 cases from the adjacent city, which is the smallest scale where the public database on COVID-19 information is available. The results of this study did not show any significant correlation between the SARS-CoV-2 levels in the plant wastewater and the COVID-19 cases in the nearest city. As the most relevant COVID-19 clinical data was from the city scale where the population was much larger than the population of the workers from the meat processing plant, it was expected that the city population failed to closely reflect the situation in the plant. Greenwald et al. [27] suggested several reasons why the wastewater signal did not significantly correlate with clinical data, including the impact of public health policy on clinical testing, the insensitivity of wastewater signal to time variation to establish any correlation, and biases with testing such that asymptomatic individuals often did not seek clinical testing, which all potentially explain why some COVID-19 cases were only detected through clinical testing but not wastewater monitoring and vice versa.

In this study, SARS-CoV-2 was consistently detected from the liquid fraction of wastewater from 9 February 2022 to 14 February 2022, which accounts for 50% of the total positive sampling days. One possible explanation for the consistent detection was that a large amount of virus was loaded in the meat processing plant as early as 9 February 2022, either from infected personnel shedding the virus or virus being washed from floors and surfaces, and the virus stayed in the tank wastewater until 14 February 2022. It is worth noting that the virus was again detected approximately a week later on 20 February 2022, which could be a result of resuspension from the sludge as a faction of the tank wastewater was emptied on Sundays in the meat processing plant. The analysis results showed that both the frequency of detection and levels of SARS-CoV-2 were higher in the solid fraction of the wastewater from the meat processing plant. This finding is consistent with the observations of Kitamura et al. [20] where they quantified SARS-CoV-2 RNAs in wastewater from wastewater treatment plants and reported consistently higher virus concentration in the solid fractions compared with the supernatant fractions. The frequency of detection in the solid fraction was approximately five times as high as in the liquid fraction during the collection period, which agrees with the study of Graham et al. [28] who found higher SARS-CoV-2 detection frequencies in the settled wastewater solids than in the corresponding influent. The SARS-CoV-2 level in the solid fraction of wastewater was up to a magnitude higher than that in the liquid fraction, with the concentrations ranging from 1.46 × 10^4^ to 1.36 × 10^5^ GCN/mL for the liquid fraction and 3.33 × 10^3^ to 1.02 × 10^6^ GCN/mL for the solid fraction. Similarly high levels of SARS-CoV-2 were found in the literature, and Peccia et al. [29] reported the virus concentrations in the sludge lead the day of positive clinical testing results by 0–2 days [30]. In a study of survivability and recovery of enveloped viruses in wastewater, Ye et al. [31] showed that coronaviruses had a higher affinity to the solid fraction of wastewater, serving as a possible explanation for the higher concentration and detection frequency of SARS-CoV-2 in the solid fraction.

The goal of this study was to provide a real-time, onsite fast screening method that can detect the presence of SARS-CoV-2 in wastewater and monitor the increase in its concentration on a daily basis using a simple molecular method. The viruses released within the plant would be continuously washed into the wastewater tank through hosing during daily operations. The RT-qPCR method can be used by trained personnel to provide results within hours, which can be used by the facility’s management to implement control measures. Although the workers at the meat processing plant were encouraged to be routinely tested, presymptomatic and asymptomatic individuals could have been present to pass on the virus, which can also be detected with the method in this study. Individual testing is required to determine which workers are infected, but a lockdown of the meat processing plant could be considered if a continuous trend of exponential increase in the SARS-CoV-2 concentration was to be observed. The meat processing plant investigated in this study has installed plastic partitions between workers in the fabrication room as a preventive measure. Body temperature can be taken with a thermometer to assist in infection control. However, asymptomatic individuals may be missed. Other preventive measures include masks, face shields, increasing spacing between workers, and disinfection of the facility when a peak in virus concentration is detected. Our study offers a cost-effective, fast, and simple method for routine monitoring for the presence and variation of the SARS-CoV-2 virus in the facility, which can assist in preventing the spread of COVID-19 and protect workers.

## 5. Conclusions

This study successfully achieved the goal of understanding the temporal variation of SARS-CoV-2 in wastewater from a meat processing plant by detecting and quantifying the viruses in wastewater over the project period. A cluster of positive results from wastewater collected in mid-February 2022 indicates that the viruses can be preserved in the solid fraction of wastewater for up to 12 days. Since only a fraction of the tank waste was treated every week and 20–30% of the waste always stayed according to the plant treatment system, the wastewater results did not accurately reflect the day-to-day change of the viruses in the meat processing plant but rather over a period of time. However, when large amounts of viruses were loaded into the tank, it was detected in the liquid fraction of wastewater in mid-February 2022. Higher SARS-CoV-2 concentrations and detection frequencies were observed in the solid fraction compared to the liquid fraction of the wastewater, possibly due to the higher affinity between the viruses and the solids. The wastewater virus level did not correlate to the city-wide COVID-19 cases, suggesting that business owners should not solely rely on public information where the scale of the city population does not match the much smaller population of the workers.

This study demonstrates that virus monitoring in wastewater at a local meat processing plant provides a promising strategy for notice of infection and virus presence at a facility-wide level, which allows for early detection and risk control in a timely manner. Although the capacity of wastewater treatment (i.e., no full turnover) hinders the detection of day-to-day change of virus level, further study should include the on-site testing results at the meat processing plant and evaluate any correlation between the wastewater-based monitoring and positive COVID-19 cases at the facility.

## Figures and Tables

**Table 1:** Detection of SARS-CoV-2 in the Liquid Fraction of Wastewater in 2022.

Sunday	Monday	Tuesday	Wednesday	Thursday	Friday	Saturday
		11 Jan	12 Jan	13 Jan	14 Jan	15 Jan
		−	−	−	−	−
16 Jan	17 Jan	18 Jan	19 Jan	20 Jan	21 Jan	22 Jan
−	−	−	−	−	−	−
23 Jan	24 Jan	25 Jan	26 Jan	27 Jan	28 Jan	29 Jan
NS	−	−	−	NS	−	NS
30 Jan	31 Jan	1 Feb	2 Feb	3 Feb	4 Feb	5 Feb
−	−	−	−	−	−	−
6 Feb	7 Feb	8 Feb	9 Feb	10 Feb	11 Feb	12 Feb
−	−	−	+	+	−	+
13 Feb	14 Feb	15 Feb	16 Feb	17 Feb	18 Feb	19 Feb
+	+	−	−	−	−	−
20 Feb	21 Feb	22 Feb	23 Feb	24 Feb	25 Feb	26 Feb
+	NS	−	−	−	NS	NS
27 Feb	28 Feb	1 Mar	2 Mar	3 Mar	4 Mar	5 Mar
−	−	−	−	−	−	−
6 Mar	7 Mar	8 Mar	9 Mar	10 Mar	11 Mar	12 Mar
−	NS	NS	NS	NS	NS	NS
13 Mar	14 Mar	15 Mar	16 Mar	17 Mar	18 Mar	19 Mar
NS	NS	NS	NS	+	+	+
20 Mar	21 Mar	22 Mar	23 Mar	24 Mar	25 Mar	26 Mar
+	NS	NS	NS	NS	NS	NS
27 Mar	28 Mar	29 Mar	30 Mar	31 Mar	1 Apr	2 Apr
NS	NS	NS	NS	−	−	−
3 Apr						
−						

Dates where the wastewater tested positive were marked with +; Dates where the wastewater tested negative were marked with −; NS means no samples collected on that date.

**Table 2:** Detection of SARS-CoV-2 in the Sludge Fraction of Wastewater in 2022.

Sunday	Monday	Tuesday	Wednesday	Thursday	Friday	Saturday
		11 Jan	12 Jan	13 Jan	14 Jan	15 Jan
		+	+	+	+	−
16 Jan	17 Jan	18 Jan	19 Jan	20 Jan	21 Jan	22 Jan
−	−	−	+	−	+	−
23 Jan	24 Jan	25 Jan	26 Jan	27 Jan	28 Jan	29 Jan
NS	−	−	−	NS	−	NS
30 Jan	31 Jan	1 Feb	2 Feb	3 Feb	4 Feb	5 Feb
−	+	+	+	+	−	−
6 Feb	7 Feb	8 Feb	9 Feb	10 Feb	11 Feb	12 Feb
−	+	+	−	−	−	+
13 Feb	14 Feb	15 Feb	16 Feb	17 Feb	18 Feb	19 Feb
+	+	+	+	+	+	+
20 Feb	21 Feb	22 Feb	23 Feb	24 Feb	25 Feb	26 Feb
+	NS	+	+	−	NS	NS
27 Feb	28 Feb	1 Mar	2 Mar	3 Mar	4 Mar	5 Mar
+	−	+	+	+	−	−
6 Mar	7 Mar	8 Mar	9 Mar	10 Mar	11 Mar	12 Mar
−	NS	NS	NS	NS	NS	NS
13 Mar	14 Mar	15 Mar	16 Mar	17 Mar	18 Mar	19 Mar
NS	NS	NS	NS	+	+	+
20 Mar	21 Mar	22 Mar	23 Mar	24 Mar	25 Mar	26 Mar
+	NS	NS	NS	NS	NS	NS
27 Mar	28 Mar	29 Mar	30 Mar	31 Mar	1 Apr	2 Apr
NS	NS	NS	NS	−	−	−
3 Apr						
−						

Dates where the wastewater tested positive were marked with +; Dates where the wastewater tested negative were marked with −; NS means no samples collected on that date.

## Data Availability

All data associated with this manuscript are available in the body of the paper and in the Supplementary Materials.

## Supplementary Materials

The following supporting information can be downloaded at: https://www.mdpi.com/article/10.3390/microorganisms11010174/s1, Data for Figure 1 and Figure 2.
